# Australian general practitioners' knowledge, attitudes and practices towards breastfeeding

**DOI:** 10.1371/journal.pone.0191854

**Published:** 2018-02-28

**Authors:** Orit Holtzman, Tim Usherwood

**Affiliations:** 1 Department of General Practice, Sydney Medical School Westmead, The University of Sydney, Sydney, NSW, Australia; 2 Hornsby Ku-ring-gai Hospital, Hornsby, NSW, Australia; TNO, NETHERLANDS

## Abstract

The aim of this study was to explore the knowledge, attitudes and practices of established general practitioners (GPs) in relation to breastfeeding. 10 GPs in the Australian Nepean Blue Mountains Health District were interviewed and the interviews transcribed and analyzed thematically. Emergent themes from each interview were identified and then compared between and across the 10 interviews. Five themes emerged following the analysis: breastfeeding knowledge and training; attitudes towards breastfeeding; GPs’ role in relation to breast feeding; GPs’ practices; influence of male gender. All the GPs interviewed had positive attitudes towards breastfeeding, however they were often lacking in knowledge and conviction to be able to provide strong support to women during their breastfeeding journey. Some reported ambivalence in their encouragement of breastfeeding due to their desire to maintain a good relationship with women who chose not to feed this way. Nine of the GPs had little or no formal breastfeeding training and relied mainly on personal experience. Their clinics did not provide formal breastfeeding support including a written breastfeeding friendly policy and most GPs were not proactive in creating such an environment. We hope that the results from this study will assist in developing breastfeeding policies and professional education to support GPs in this role.

## Introduction

Beyond the benefits that breastfeeding confers to the mother-child relationship, breastfeeding lowers the incidence of many childhood illnesses, such as middle ear infections, diarrhea, respiratory infections, sudden infant death syndrome, diabetes mellitus and malocclusion, and is associated with increased intelligence in the child[1, 2]. In the mother, breastfeeding has been shown to decrease the frequency of breast cancer [2, 3], ovarian cancer and endometrial cancer [2, 4, 5], as well as facilitating weight loss [6], providing protection against type 2 diabetes [2] and conferring temporary contraception[2]. The World Health Organization (WHO) defines breastfeeding as the baby receiving any breast milk, even if only once;, and, exclusive breastfeeding as when the infant receives only breast milk and no other liquids or solids with the exception of drops or syrups consisting of vitamins, mineral supplements or medicines [7]. A recent meta-analysis by Victora et al [2] showed that exclusively breastfed infants in moderate to low income countries had only 12% of the risk of death compared with those who were not breastfed. In children aged 6–23 months, any breastfeeding was associated with a 50% reduction in deaths. In high-income countries, breastfeeding was associated with a 36% reduction in sudden infant deaths and a 58% decrease in necrotising enterocolitis. The authors concluded that "The scaling up of breastfeeding to a near universal level could prevent 823000 annual deaths in children younger than 5 years and 20000 annual deaths from breast cancer".

Infant Feeding Guidelines recommend exclusive breastfeeding until around 6 months of age when solid foods are introduced, and continued breastfeeding until 12 months and beyond [7]. Studies have shown that breastfeeding initiation rates [8–10] and breastfeeding duration [10, 11]) increase if a doctor provides information, support, and encouragement. General practitioners (GPs) are in ideal position to support and encourage breastfeeding.

However, in order to be able to provide support and encouragement, GPs need to have positive attitudes towards breastfeeding and also to possess the appropriate knowledge to be able to provide guidance and assist in solving breastfeeding problems. Unfortunately, studies from around the world repeatedly show that many health care professionals have significant knowledge deficits that impair their ability to support and assist breastfeeding mothers and their infants [1, 12–18]. In Australia, Amir and Pirotta [19] found that GPs in Victoria lacked knowledge about the safe use of medicines in breastfeeding women. Brodribb et al [20] found that GP registrars (ie. trainees) Australia-wide, lacked knowledge concerning the treatment of breastfeeding-related conditions. Importantly, a large proportion of participants in both studies viewed breastfeeding and formula feeding as almost equal in their benefits [19, 21].

Little is known about the attitudes and practices of fully qualified general practitioners (GPs) in Australia towards breastfeeding [22]. We therefore set out to explore the knowledge, attitudes and practices of fully qualified GPs in relation to breastfeeding.

## Method

This qualitative study used semi-structured, in-depth interviews with a small, purposive sample of fully qualified GPs in established clinical practice.

### Sample and setting

We sent a letter by mail to all 448 GPs in the Nepean Blue Mountains health district of New South Wales inviting their participation. Out of those, 8 replied, expressing their interest in participating in the study, and 2 more were recruited by snowballing. The sample did not seek to be representative but to allow us to explore the variety of knowledge, attitudes and practices across the study population.

### Interviews and transcription

All the GPs interviewed signed a consent form allowing the researchers to use the information obtained in the interviews. Semi structured interviews of 30–45 minutes (see S1 Interview Guide) were conducted and recorded by OH, a female researcher who was a medical student at the time, in the GPs’ offices between December 2014 and February 2015. Demographic information was also collected from 9 of the 10 participants (see Table 1). The 10^th^ participant did not provide this information to the researchers.

**Table 1:** Demographics of sample (N = 10).

Characteristic	Number or Median (Range)
Females	7
Males	3
Age (N = 9, see note)	44 (31–66) years
Time in practice (N = 9, see note)	13 (4–38) years

**Note**. Information not provided by one participant.

The interviews were transcribed. OH undertook their initial coding, then worked with her supervisor TU to refine the codes and identify emergent themes. By the 10^th^ interview no new themes emerged and thus no further participants were sought out, despite the small sample. Repeat interviews were not done and the participants did not review the transcripts. However, the researchers compared the transcripts to the taped interviews to ensure fidelity of the transcripts. NVivo software was used to manage the data and assist with the coding process.

### Analysis

**Fig 1:**
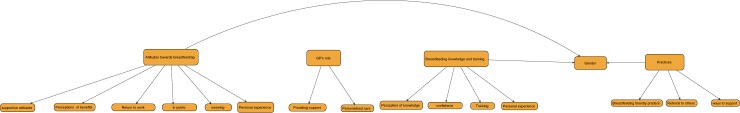
Coding tree.

Our analytic aim was to describe participants’ knowledge, attitudes and practices, so we adopted a simple thematic approach to analysis of the interviews. As described by Braun and Clarke (2006), thematic analysis is a method for identifying, analyzing and reporting patterns (themes) within data, where the researcher codes relevant utterances by participants (i.e. those relating to knowledge, attitudes and practices in relation to breastfeeding) in the data and then collates these codes into subthemes and overarching themes [23]. The coding tree (Fig 1) illustrates those themes and subthemes, and their relationships.

This work was approved by the Office of Research Integrity at the University of Sydney ethics reference number 2014/422.

## Results

Ten GPs agreed to participate in the study (7 females and 3 males, see Table 1).

Five themes emerged from the analysis: breastfeeding knowledge and training; attitudes towards breastfeeding; GPs’ role in relation to breast feeding; GPs’ practices; influence of male gender. Table 2 lists the five themes and their subthemes, and Fig 1 illustrates their relationships.

**Table 2:** Themes and subthemes.

THEMES	SUBTHEMES
Breastfeeding knowledge and training	Perception of knowledge Confidence Training Personal experience
Attitudes towards breastfeeding	Supportive attitudes Perceptions regarding benefits Return to work Breastfeeding in public Weaning Personal experience
GPs’ role in relation to breastfeeding	Providing support Personalized care
GPs’ practices	Breastfeeding friendly clinics Ways to support Referral to others
Influence of male gender	Male gender and attitudes Male gender and knowledge Male gender and practices

### 1. Breastfeeding knowledge and training

The first theme (Table 2) identified was the GPs’ breastfeeding knowledge and training. It contained four subthemes: perception of knowledge; confidence; training; personal experience.

#### 1.1 Perception of knowledge

Most of the GPs perceived their knowledge as moderate and possibly in need of updating:

GP 1 (female): Look, it's probably poor now. It was probably at my best about 10 years ago because my patients grow older with me, so my patients are now out of breastfeeding. So it probably has diminished

However, some were more confident and noted the availability of expert advice when needed:

GP 2 (male): It works for me. I mean I don't know, kind of how long is a piece of string? If I don't know something, I'll either Google it, in front of my client, or pick up a phone and talk to a colleague. I'm happy with my level of understanding…

#### 1.2 Confidence

Nevertheless, all the GPs felt fairly confident managing the breastfeeding issues they encountered in general practice. While most of them felt they could manage medical issues such as mastitis themselves, they stated they would refer their patient to a lactation consultant for more complex issues, such as latching and supply.

GP 3 (female): If it is was mastitis or a fungal infection that sort of thing, I'd be confident handling that, but if it was to do with attachment [latching] or those more, yeah I'd rely more heavily on a lactation consultant for that sort of stuff, yeah.

#### 1.3 Training and personal experience

Surprisingly, most of the GPs stated that they did not receive any training in management of breastfeeding throughout their medical degree and GP training.

GP 2 (male) No there wasn't any. No going right back—gee going back a while here—I did my obstetric training….. I'm just scanning across to all of our lecturers there and no, breastfeeding didn't even rate a mention…I did postgrad work over in….., again, breastfeeding, no it didn't rate a mention.

A couple of GPs were able to recall one day of training and one had spent a term in an obstetric hospital where they had a lot of exposure to breastfeeding women. Some had sought out further education:

GP 4 (female): I've certainly done extra study off my own back because I feel that the learning that we're given in medical school is appalling on breastfeeding.

And others mentioned their own or their partner’s experience:

GP 2 (male): I've learnt it probably through my marriage, wife, and just from what I've observed.

### 2. Attitudes towards breastfeeding

The GPs’ attitudes towards breastfeeding were grouped into six subthemes: supportive attitudes; perceptions of breastfeeding advantages and benefits; return to work; breastfeeding in public; weaning; personal experience (Table 2).

#### 2.1 Supportive attitudes

The GPs viewed breastfeeding positively and some took an evolutionary perspective:

GP 5 (male): My views towards breastfeeding, human beings are genetically evolved into breastfeeding their neonates. It's natural, normal and potentially very beneficial part of human life.

However, they also noted the importance of respecting and supporting mothers’ individual circumstances and choices:

GP 6 (female): ….I guess for people who can't breastfeed or don't want to breastfeed I try to support that because I don't want to be putting anyone—I guess I want to maintain my relationship with them and be able to support them in their decision but I support breastfeeding as much as I can.

#### 2.2 Perceptions regarding benefits

Most GPs considered breastfeeding as having health and psychological benefits, as well as being nutritionally superior to formula feeding and more convenient and cheap.

GP 2 (male):…That's huge, enormous, on so many levels, physical, emotional,psychological…

One GP noted a common misconception about a possible detrimental effect of breastfeeding on the infant’s health:

GP 7 (male): I suppose there's this other thing about breast fed babies getting more constipated possibly due to less fluid intake. As in you might get more water from the formula milk. But I've not had huge problems with that with my mums who breast feed….

In contrast, a few GPs noted potential risks with formula:

GP 8 (female): I guess if the mother perhaps doesn't understand the instructions properly or can't afford to buy more formula, she might start watering it down so it's not ideally mixed. You know, it could be stored too long and go off, those kind of issues are possibilities.

All but one perceived breastfeeding as superior to formula feeding in promoting bonding between the mother and infant:

GP 5 (male):….there's not the lips to skin sort of contact. So it's a different interaction between a mother and baby.

GP 4 (female): I just think it's amazing for attachment [bonding], for bonding as well as a source of perfect nutrition…

However, one GP was quite ambivalent in their view of how breastfeeding affects bonding:

GP 3 (female): I think it's probably good if it's going well. If it's not going well it would be stressful…. I think bonding is quite complex and there's a lot more to it than just how you feed.

Interestingly, in regards to views on how breastfeeding affects bonding between the father and baby, while the male GPs who were fathers had a positive view, some of the female GPs were concerned that breastfeeding might make fathers feel excluded, whereas bottle feeding could help them feel more involved.

GP 4 (female): Look, I think that, you know, sometimes fathers can feel a little bit left out by breastfeeding, because it's, you know, it's not something they can generally, obviously, do.

GP 8 (female): I think a lot of men would find there being some element of envy or something that that is something they cannot do, that they’ll never do with their child, and that they're denied that complete intimacy with the child. I think that might be an issue as well.

#### 2.3 Breastfeeding and return to work

All GPs were very supportive of mothers continuing breastfeeding when they return to work:

GP 7 (male): Oh absolutely, yeah. I mean I think a responsible society should support as many options as possible.

GP 6 (female): I think ideally if the woman wishes to continue to be able to breastfeed when she returns to work that there should be somewhere that's available for her.

However, they also noted it as often very challenging for the woman, especially if the work place was not supportive. One GP who was also a breastfeeding mother, had an interesting example of how the work place can make a difference:

GP 4 (female): For instance, when I started here my 12 month old used to come in at lunchtime and have a feed. Whereas when I was in the hospital system, it was just awful. It was impossible.

#### 2.4 Breastfeeding in public

All the GPs had a positive view of breastfeeding in public, perceiving it as a natural thing and socially necessary:

GP 4 (female): I think breastfeeding in public is wonderful, because it makes people aware and normalizes breastfeeding, because breastfeeding is a normal way to feed a child….I work with teenagers with Youth Off the Streets and unfortunately a lot of them have had pregnancies, you know, aged 13, 14 and none of them breastfeed because they see that as disgusting because it's sexualised. They see the breasts as sexual.

#### 2.5 Weaning

The GPs interviewed felt that the decision to wean was an individual one:

GP 4 (female): I think weaning is a matter for the mother and the baby and when they both want to wean then they can wean.

Only one GP mentioned the Australian National Health and Medical Research Council (NHRMC) guidelines which recommend exclusive breastfeeding up to 6 months and continuation of breastfeeding for at least 12 months. One GP stated that he recommends mothers to wean if the baby bites while breastfeeding, which can happen well before the recommended twelve months:

GP 5 (male):…the final weaning takes place when the baby develops teeth. Numerous mothers say, ouch when he or she started biting and I said well, that's enough, and breastfeeding was stopped.

Quite a few GPs mentioned extended breastfeeding (beyond the age of 2 years) and had varied views towards it.

Some were supportive:

GP 2 (male): I know of some mums who are breastfeeding their kids when they're three or four. My point is, so what? That's what works for them.

Others had quite negative views about extended breastfeeding and some perceived it as something that is done due to the mother’s wishes and does not really benefit the child and could even cause psychological harm, especially if extended beyond the of three or four:

GP 5 (male): I don't think there's any kind of physiological reason. Usually it fulfils something which is a belief pattern or a philosophical pattern in the mother, predominantly.

GP 1 (female): But some patients are still breastfeeding their children at age six or seven. I find that a bit strange and I actually worry about the child's peers. That is not the norm and I wonder how much—who's benefitting the most from that and I suspect it's the mother rather than the child.

#### 2.6 Personal experience

Most GPs, both female and male, had a positive personal experience with breastfeeding, either from themselves breastfeeding, their partner breastfeeding, or family and friends’ breastfeeding experiences. Positive experiences contributed to positive attitudes:

GP 4 (female): ….I've grown up in a family of women who have breastfed and it's just very normal. So I've always grown up just seeing breastfeeding as a normal part of having a child.

GP 6 (female): I guess potentially in terms of the people around me, in terms of friends and family are all quite supportive of breastfeeding so that potentially influences my views.

One female GP who had no personal experience with breastfeeding, felt that her lack of experience contributed to her neutral attitude towards breastfeeding:

GP 9 (female):..*I think it does because I think my attitude at this stage is still*, *well if you can't breastfeed or choose not to breastfeed for whatever reason that's still okay and it's still a choice*, *so long as it's an informed choice*. *Maybe if I have had more experience in the area that may change*, *because I still think I'm very open to all options*, *whether that be breastfeeding or formula feeding or whatever…*

If a GP had a negative personal experience, this could affect their views:

GP 8 (female): So yeah, that has strongly influenced my attitude because I think I was healthy, I did everything right. I followed every piece of advice. I had recurrent mastitis and I was a mess….So I had a lot of problems with it, so I'm very skeptical when people say every woman can breastfeed and it's sort of, well, you mightn't if you can't.…

### 3. GPs’ role in relation to breast feeding

The third theme identified was the GP’s role in relation to breastfeeding women. The theme contained two subthemes: providing support and personalized care.

#### 3.1 Providing support

Most GP’s felt that they had an important role in supporting and encouraging breastfeeding. They noted that this support needs to start during pregnancy and that as family doctors they have particular authority and responsibility in this regard.

GP 1 (female): I think we're essential. We're the people that know their story. We're the people that have looked after them, often looked after a generation of their family.

GP 10 (female): I think a GP if they were discouraging of breastfeeding would hold a lot of power over that woman. I think vice versa they have a lot of influence over the woman if they’re encouraging of them too.

However, one GP felt that his role in supporting breastfeeding was minimal.

GP 2 (male): If someone asks me about breastfeeding, my first thought is, well why are they asking me, what would I know about that? …fairly simply, say as little as possible. I don't think it's particularly my business, except to encourage it as a component of good health.

#### 3.2 Personalized care

A common theme that emerged from the interviews was the GPs’ perception of their role in providing personalized care. This seemed to be a key guiding principle for many of the GPs.

GP 3 (female): I just see my role is to support the woman and what's working well for her in her life and in the big picture of her life

GP 7 (male):…it's based upon the person in front of me. So it's a personalized medical service. It's personalized care, I think.

### 4. GPs’ practices

The fourth theme that emerged from the analysis of the interviews included in this study was GPs’ practices in regards to breastfeeding women. This theme contained three subthemes: breastfeeding friendly clinics; ways to support; and referral to others.

#### 4.1 Breastfeeding friendly clinics

Most of the GPs regarded their clinics as “breastfeeding friendly” in the sense that the doctors were supportive of breastfeeding and that breastfeeding in the waiting room and during consultations was acceptable and even encouraged.

GP 5 (male): I would think that most, if not all of the doctors would, in some way, proactively discuss or encourage breastfeeding. …certainly breastfeeding is allowed and encouraged in the waiting room.

GP 4 (female): All of our staff understands that breastfeeding is completely okay out in the waiting room, you know, anywhere a woman likes basically.

However, despite their positive attitudes, most of the GPs were ambivalent about making this explicit in the waiting room, and did not have a dedicated breastfeeding area for women who wished to have more privacy.

GP 5 (male): We don't have a sign saying, breastfeeding in this waiting room is encouraged because we've got lots of other signs. I think that's a bit over the top

GP 9 (female): We haven't. On the occasions that women have wanted to breastfeed we offer them a private area, which is usually one of the other rooms to breastfeed…We don't have any signs up saying, it's okay to breastfeed. I certainly haven't seen any women in the waiting room breastfeeding….

#### 4.2 Ways to support

The main way most GP’s supported breastfeeding women was by providing information:

GP 5 (male): ….provision of information through books, pamphlets, associations…

GP 4 (female): ….giving them information. I'll often, when they're pregnant, I'll give them information about the breastfeeding associations, breastfeeding education classes. I offer them information from myself. I have posters in my room about breastfeeding.

The GPs also emphasized the importance of being open to discuss any concerns a woman might have in regards to breastfeeding, being empathetic and nonjudgmental, and providing medical assistance if needed.

GP 5 (male): When a woman presents with pregnancy I will go and not only discuss matters related to the pregnancy, but what happens thereafter. Is she planning to take a long time off, a short time off? Is she planning to breastfeed? What the issues are around that.

GP 1 (female): I think what general practice is very good at is adapting the consultation to where the patient is and often because you've seen them prenatally you know their story. So you know where they are in life and can structure the consultation to sort of meet their agenda and hopefully put some breastfeeding tips in there at the same time.

#### 4.3 Referral to others

Most of the GPs interviewed in this study used referral to lactation consultants and support groups, as part of their support of breastfeeding women.

*GP 4 (female)*: *I'll give them information about the breastfeeding associations*, *breastfeeding education classes…*.*if it's attachment* [latching] *issue*, *which perhaps might need a lactation consultant*, *I'll send them on to them*. *I might refer them onto the breastfeeding helpline or the breastfeeding site…*.

### 5. Influence of male gender

The final theme that emerged was the way a GP’s gender influences their attitudes, knowledge and practices towards breastfeeding.

#### 5.1 Male gender and attitudes

Two of the three male GPs stated they had positive attitudes towards breastfeeding while the third described his views not as “positive” but “liberal”, by which he explained that while he was supportive of breastfeeding, as a doctor it was not his place to form a view on his patients’ choices:

GP 2 (male): In one word, liberal…….I've observed over the years attitudes, how conditions and structures appear to have been placed on breastfeeding.……The most successful and happy content couples, meaning mother and baby, are those who basically run their own race….

#### 5.2 Male gender and knowledge

Male GPs in this study did not see themselves as less knowledgeable than their female colleges, and like the female GPs the two male GPs who were parents felt that much of their knowledge came from personal and clinical experience:

GP 5 (male): A lot of the tricks of the trade….. have come about from my experience, my wife's experience and other patients' experience… I wouldn't claim to be an expert, but I feel quite happy and confident to either discuss all these issues with my patients or turn them to someone…

#### 5.3 Gender and practices

The male GPs interviewed in this study felt comfortable discussing breastfeeding issues with their female patients:

GP 7 (male): I don't feel uncomfortable talking about breast feeding. In fact I probably like these more challenging issues….

One male GP though, emphasized the point that male GPs should be careful with trying to influence a woman’s decision to breastfeed, as they have no direct experience with the issue:

GP 2 (male): So I would hope that doctors generally take a fairly liberal approach to the whole thing and frankly don't meddle in something of which they know nothing. Unless they're females who have breastfed themselves and raised kids. They're the people—if advice is offered, I'd like to think that it came from them.

Another male GP thought that some female patients may prefer discuss breastfeeding issues with a female doctor:

GP 5 (male): Occasionally women will say, well you don't know how it feels and I will say, yes I don't know how it feels…..I know there are some women who won't talk to me about it because they assume as a man I know very little.

Most of the female GPs thought that while female GPs have a better ability to empathize, especially if they have breastfed themselves, GPs’ ability to support their breastfeeding patients was more related to their relationship with the patient, rather than to gender.

GP 4 (female): I suppose some women would feel more comfortable with a female……if the GP is comfortable then they will be comfortable too. I think male GPs still have a very important role to play.

GP 1 (female): I think it's much more to do with the relationship rather than the gender

## Discussion

In our study of the knowledge, attitudes and practices of established general practitioners in relation to breastfeeding, we identified five themes: breastfeeding knowledge and training; attitudes towards breastfeeding; GPs’ role in relation to breast feeding; GPs’ practices; and, influence of male gender.

In relation to the first theme, most of the GPs felt that their knowledge was moderate at best and could be improved. They reported that they had received almost no formal breastfeeding training in medical school or subsequently. Their main source of knowledge and confidence was personal experience. The same picture has been painted by previous research. Studies from around the world repeatedly show that health care professionals have significant knowledge deficits that impair their ability to support breastfeeding [1, 13–18]. In Australia, Amir and Pirotta [19] found that GPs in Victoria lacked knowledge about the safe use of medication in breastfeeding. Brodribb et al [20] found that GP registrars (trainees) Australia-wide lacked knowledge concerning the treatment of breastfeeding-related conditions. Interestingly, all the GPs interviewed in our study felt fairly confident managing the breastfeeding issues they encounter. This is in contrast to Brodribb’s [21] findings in her study of Australian GP registrars, where some felt that their skills and knowledge were inadequate to provide competent care, and expressed concern that they would not be able to manage problems that they had not previously came across personally or professionally. This difference could be explained by the fact that the fully qualified GPs interviewed in this study had more personal and professional breastfeeding experience as compared with the trainees. However, even amongst the GPs interviewed in the current study, some of this confidence seems to have come from their ability to refer to a lactation consultant for the management of more complex issues, such as latching and supply. A recent study found that experienced GPs in Tasmania were not concerned by their level of breastfeeding knowledge but by barriers to collaborating with other health practitioners [22].

In relation to the second theme, attitudes towards breastfeeding, all the GPs who participated perceived breastfeeding as positive, describing it as the natural choice and the gold standard. They were supportive of breastfeeding while returning to work and towards breastfeeding in public. This is very much in line with previous studies that have found health care professionals from the United States, Mexico, Ireland, Scotland, England, Israel, Taiwan and Iraq demonstrate positive breastfeeding attitudes [12, 15–18, 24]. In Australia, GPs and GP trainees were also found to have positive attitudes towards breastfeeding [20, 21, 25, 26]. However, despite these positive attitudes, some interviewed GPs were ambivalence in their encouragement of breastfeeding, mainly due to their desire to maintain a good relationship with patients who chose not to, or were unable to breastfeed. Only one GP noted that it is rare for a woman to be completely unable to breastfeed for physiological reasons [3]. Brodribb et al. [26] described similar ambivalence amongst Australian GP trainees, also attributed to their wish to support or remain neutral regarding their patients’ infant feeding methods. Moreover, in a questionnaire received from 161 Australian GP trainees, nearly half thought that breastfeeding and formula-feeding were both equally acceptable methods of infant feeding [26]. While it is understandable that GPs wish to support to their patients above all, caution is needed so that the GP’s view of breastfeeding is not perceived as neutral; it has been demonstrated in several studies that breastfeeding intention, initiation and duration rates are similar if a woman’s health care practitioner does not express an opinion, or has negative views about breastfeeding [27, 28].

Lack of, or negative personal experience with breastfeeding also contributed to ambivalence. Previous studies have shown that doctors with breastfeeding experience had more positive attitudes towards breastfeeding [16, 29, 30]. Moreover, this effect seems to depend on the length of breastfeeding experience, as Australian GP trainees with more than 52 weeks of personal breastfeeding experience (self or partner) were found to have more positive breastfeeding attitudes than registrars with less personal experience [25].

Regarding the health benefits of breastfeeding, all GPs viewed breastfeeding as beneficial for the infant’s health, but only one mentioned health benefits for the mother (in the form of weight loss). However, studies have shown that breastfeeding decreases the incidence of many childhood illnesses [1, 2]. In the mother, breastfeeding has been shown to decrease the risk of breast cancer [2, 31], ovarian cancer and endometrial cancer [2, 4, 5], as well as facilitating weight loss [6], protecting against type 2 diabetes and conferring temporary contraception [2]. This deficit in knowledge of fully trained, practicing GPs may impair their ability to support breastfeeding women.

Breastfeeding was also perceived as convenient and cheap by all GPs interviewed, and safer as there is less room for error than in the preparation of baby formula. In comparison, in a study conducted by Brodribb et al. [20] only 63% of Australian GP trainees rated breastfeeding as more convenient than formula feeding. It is possible that the more experienced GPs interviewed in the current study, had been exposed to a larger number of breastfeeding women and thus were convinced by the apparent ease of breastfeeding.

All but one GPs interviewed perceived breastfeeding as superior to formula feeding in promoting bonding between the mother and infant. The one remaining GP suggested that if there are breastfeeding difficulties it can be detrimental to the mother-infant bond. These results are similar to those of Brodribb [20], that 91.3 percent of GP trainees agreed that breastfeeding increases mother–infant bonding. In contrast, only 77% of midwives in Scotland rated positively the effect of breastfeeding on mother–infant bonding [32]. These differences may be attributed to the population sampled.

Regarding bonding between the father and baby, while the male GPs who were fathers themselves had a positive view, viewing breastfeeding as a three way relationship, some of the female GPs were concerned that breastfeeding might make fathers feel excluded, in contrast to their involvement through bottle feeding. In the study by Brodribb et al [20], 80% of GP trainees disagreed with the statement “*Fathers feel left out if a mother breastfeeds*” with no significant difference between male and female respondents (81.85% vs. 79.25%): the difference might come from personal experience of the female GPs in the current study.

All GPs interviewed were supportive of breastfeeding following return to work, however they perceived it as challenging, especially if the workplace is not supportive. Nevertheless, most of the GPs had either breastfed themselves while working, had a partner who breastfed, or witnessed family and friends breastfeeding successfully while returning to work. Of note, research suggests that women’s passion and intention may be more important in determining their likelihood of breastfeeding than the actual work environment [33].

The WHO recommends exclusive breastfeeding for the first 6 months of life, with continued breastfeeding and addition of appropriate complementary foods up to 2 years of age and beyond, for as long as the mother and child wish [34]. In Australia, the national Infant Feeding Guidelines recommend exclusive breastfeeding until around 6 months of age when solid foods are introduced, and continued breastfeeding until 12 months and beyond [7]. While the rates of breastfeeding in Australia at discharge from hospital are high and rising, from 87% in 2001 to 95.9% in 2010 [35, 36], there is a substantial decline in breastfeeding rates in the following weeks and months. In 2010, breastfeeding rates dropped to 75% at 2 months, to 69% at 4 months and to 42.2% at 7–12 months. Exclusive breastfeeding rates were even lower, with only 48% of infants being exclusively breastfed from birth to 2 months [35]. The GPs interviewed in the study felt that their main role was providing support and personalized care to their patients, supporting them in whatever infant method decision they would make. Most of the GPs did not feel that it was their role to influence the infant feeding decision making process. Brodribb et al. [37] found similar perceptions amongst Australian GP registrars, who felt that “*The GP was not expected to sway the mother’s decision making process beyond providing information*”. However, the evidence shows that women whose doctor encourages them to breastfeed, are more likely to initiate and continue breastfeeding [9, 10]. Finneran & Murphy found that GPs who had formal training in breastfeeding were more likely to promote the practice [17].

Most of the GPs interviewed in this study used resources such as brochures and websites and referral to lactation consultants and support groups to support breastfeeding women. Referral for the management of more complicated management issues such as latching and supply is a common finding practice in Australia and around the world [37, 38]. While GPs cannot be expected to possess expert knowledge in everything, as Brodribb notes [37], it is important for GPs to at least be able to initially assess breastfeeding problems as well as refer appropriately. This gives further support to the findings of previous studies which concluded that GPs would benefit from targeted breastfeeding education [21, 22].

The Academy of Breastfeeding Medicine has developed a protocol for creating a breastfeeding friendly physician’s office [39], “*enthusiastically promote[ing] and supporting breastfeeding through the combination of a conductive office environment and education of healthcare professionals*, *office staff and families*”. A key recommendation is to establish a written breastfeeding friendly clinic policy and provide copies to all professionals working within the clinic. In the current study, only one GP stated that their clinic had a written breastfeeding-friendly practice policy and none of the GPs had actually read such a policy. Most of the GPs regarded their clinic as “breastfeeding friendly” in the sense that the doctors and staff were supportive and that breastfeeding in the waiting room and during consultations was acceptable and even encouraged. However, despite their support for breastfeeding, most of the GPs were unsure of whether any supportive signs were placed in the clinic, and most of the practices did not have a dedicated breastfeeding area for women who wished to have more privacy.

The final theme that emerged in this study is the effect of male GPs’ gender on their knowledge, attitudes and practices. The males in this study did not evaluate themselves as less knowledgeable than their female colleagues, rating their knowledge moderate to good, and like the female GPs the two male GPs who were parents felt that most of their knowledge came from personal experience. Previous studies have shown inconsistent results regarding the influence of doctors’ gender on confidence and knowledge. While some studies show no significant difference between genders [12, 40], others have found female doctors to be more confident than male doctors [41, 42]. In a study of rural GP trainees in Australia, registrars perceived that women thought female doctors to be more knowledgeable and skilful concerning breastfeeding, regardless of their training or experience [43]. In contrast, in the current study, while acknowledging that some female patients would prefer to discuss breastfeeding issues with female doctors, the male GPs felt comfortable and capable of managing breastfeeding issues.

A limitation of our study is that it drew a small sample of GPs from a limited geographical area. Thus, it may not be representative of all GPs. This is especially true in regards to male GPs, as only 3 responded to the invitation letter sent. Nevertheless, due to the limited evidence in the specific area of breastfeeding knowledge, attitudes and practices of experienced GPs in Australia in the past 20 years (22), the study gives new insight.

While all the GPs interviewed had positive attitudes towards breastfeeding, they were often lacking in knowledge and conviction to provide strong support for women during their breastfeeding journey. This is not surprising considering most GPs had little or no formal breastfeeding training and relied mostly on personal experience. The GPs’ clinics did not provide formal breastfeeding support in terms of a written breastfeeding friendly policy, and most GPs were not proactive in creating such an environment. It has been found that breastfeeding education for GPs result in greater knowledge, improved use of resources, and a more proactive approach to breastfeeding support and the creation of a breastfeeding-friendly environment [44, 45]. Such education should also address the ambivalence that some GPs report in their encouragement of breastfeeding, due to their desire to maintain a good relationship with women who chose not to feed this way. Previous research has demonstrated that breastfeeding intention, initiation and duration rates are similar if a woman’s health care practitioner does not express an opinion, or has negative views about breastfeeding [27, 28]. We hope that the results from this study will assist in developing breastfeeding policies and professional education to support GPs in this role.

## Supporting information

S1 Interview Guide (DOCX)

## References

[1] Guise JM, Freed G. Resident physicians' knowledge of breastfeeding and infant growth. Birth. 2000;27(1):49–53. 10865561 10.1046/j.1523-536x.2000.00049.x

[2] Victora CG, Bahl R, Barros AJ, Franca GV, Horton S, Krasevec J, et al Breastfeeding in the 21st century: epidemiology, mechanisms, and lifelong effect. Lancet. 2016;387(10017):475–90. doi: 10.1016/S0140-6736(15)01024-7 26869575 10.1016/S0140-6736(15)01024-7

[3] Newcomb PA, Storer BE, Longnecker MP, Mittendorf R, Greenberg ER, Clapp RW, et al Lactation and a reduced risk of premenopausal breast cancer. The New England journal of medicine. 1994;330(2):81–7. doi: 10.1056/NEJM199401133300201 8259187 10.1056/NEJM199401133300201

[4] Rosenblatt KA, Thomas DB. Prolonged lactation and endometrial cancer. WHO Collaborative Study of Neoplasia and Steroid Contraceptives. International journal of epidemiology. 1995;24(3):499–503. 7672888 10.1093/ije/24.3.499

[5] Rosenblatt KA, Thomas DB. Lactation and the risk of epithelial ovarian cancer. The WHO Collaborative Study of Neoplasia and Steroid Contraceptives. International journal of epidemiology. 1993;22(2):192–7. 8505173 10.1093/ije/22.2.192

[6] Dewey KG, Heinig MJ, Nommsen LA. Maternal weight-loss patterns during prolonged lactation. The American journal of clinical nutrition. 1993;58(2):162–6. 8338042 10.1093/ajcn/58.2.162

[7] World Health Organization. Indicators for assessing infant and young child feeding practices. Geneva, Switzerland: World Health Organization 2007 Available from: www.who.int/nutrition/publications/iycf_indicators_for_peer_review.pdf

[8] Bentley ME, Caulfield LE, Gross SM, Bronner Y, Jensen J, Kessler LA, et al Sources of influence on intention to breastfeed among African-American women at entry to WIC. Journal of human lactation: official journal of International Lactation Consultant Association. 1999;15(1):27–34. 10578772 10.1177/089033449901500109

[9] Lu MC, Lange L, Slusser W, Hamilton J, Halfon N. Provider encouragement of breast-feeding: evidence from a national survey. Obstetrics and gynecology. 2001;97(2):290–5. 11165597 10.1016/s0029-7844(00)01116-9

[10] Li L, Zhang M, Scott JA, Binns CW. Factors associated with the initiation and duration of breastfeeding by Chinese mothers in Perth, Western Australia. Journal of human lactation: official journal of International Lactation Consultant Association. 2004;20(2):188–95. 15117518 10.1177/0890334404263992

[11] Taveras EM, Capra AM, Braveman PA, Jensvold NG, Escobar GJ, Lieu TA. Clinician support and psychosocial risk factors associated with breastfeeding discontinuation. Pediatrics. 2003;112(1 Pt 1):108–15. 12837875 10.1542/peds.112.1.108

[12] Williams EL, Hammer LD. Breastfeeding attitudes and knowledge of pediatricians-in-training. American journal of preventive medicine. 1995;11(1):26–33. 7748583

[13] Freed GL, Clark SJ, Sorenson J, Lohr JA, Cefalo R, Curtis P. National assessment of physicians' breast-feeding knowledge, attitudes, training, and experience. JAMA. 1995;273(6):472–6. 7837365 10.1001/jama.1995.03520300046035

[14] Burglehaus MJ, Smith LA, Sheps SB, Green LW. Physicians and breastfeeding: beliefs, knowledge, self-efficacy and counselling practices. Canadian journal of public health. 1997;88(6):383–7. 9458564 10.1007/BF03403911 PMC6990161

[15] Nakar S, Peretz O, Hoffman R, Grossman Z, Kaplan B, Vinker S. Attitudes and knowledge on breastfeeding among paediatricians, family physicians, and gynaecologists in Israel. Acta paediatrica. 2007;96(6):848–51. doi: 10.1111/j.1651-2227.2007.00310.x 17537013 10.1111/j.1651-2227.2007.00310.x

[16] Ingram J. Multiprofessional training for breastfeeding management in primary care in the UK. International breastfeeding journal. 2006;1(1):9 doi: 10.1186/1746-4358-1-9 16722540 10.1186/1746-4358-1-9 PMC1475559

[17] Finneran B, Murphy K. Breast is best for GPs-or is it? Breastfeeding attitudes and practice of general practitioners in the Mid-West of Ireland. Irish medical journal. 2004;97(9):268–70. 15568583

[18] Al-Nassaj HH, Al-Ward NJ, Al-Awqati NA. Knowledge, attitudes and sources of information on breast feeding among medical professionals in Baghdad. Eastern Mediterranean health journal. 2004;10(6):871–8. 16335775

[19] Amir LH, Pirotta MV. Medicines for breastfeeding women: a postal survey of general practitioners in Victoria. The Medical journal of Australia. 2009;191(2):126 19619105 10.5694/j.1326-5377.2009.tb02712.x

[20] Brodribb W, Fallon A, Jackson C, Hegney D. Breastfeeding and Australian GP registrars-their knowledge and attitudes. Journal of human lactation: official journal of International Lactation Consultant Association. 2008;24(4):422–30. 18974291 10.1177/0890334408323547

[21] Brodribb W, Fallon T, Jackson C, Hegney D. Attitudes to infant feeding decision-making-a mixed-methods study of Australian medical students and GP registrars. Breastfeeding review: professional publication of the Nursing Mothers' Association of Australia. 2010;18(1):5–13. 20443434

[22] Ayton J, Howes F, Hansen E, Nelson M. Evaluating the prevention of premature cessation of exclusive breastfeeding in the general practice setting during the scheduled child immunisation consultation: a pilot study. Australian journal of primary health. 2015;21(3):299–304. doi: 10.1071/PY13152 24898802 10.1071/PY13152

[23] Braun V, Clarke V. Using thematic analysis in psychology. Qualitative Research in Psychology. 2006;3(2):77–101.

[24] Anchondo I, Berkeley L, Mulla ZD, Byrd T, Nuwayhid B, Handal G, et al Pediatricians', obstetricians', gynecologists', and family medicine physicians' experiences with and attitudes about breast-feeding. Southern medical journal. 2012;105(5):243–8. doi: 10.1097/SMJ.0b013e3182522927 22561534 10.1097/SMJ.0b013e3182522927

[25] Brodribb W, Fallon A, Jackson C, Hegney D. The relationship between personal breastfeeding experience and the breastfeeding attitudes, knowledge, confidence and effectiveness of Australian GP registrars. Maternal & child nutrition. 2008;4(4):264–74. 18811791 10.1111/j.1740-8709.2008.00141.x PMC6860797

[26] Brodribb W, Fallon AB, Jackson C, Hegney D. Breastfeeding knowledge—the experiences of Australian general practice registrars. Australian family physician. 2009;38(1–2):26–9. 19283232

[27] Counsilman JJ, Mackay EV, Copeland RM. Bivariate analyses of attitudes towards breast-feeding. The Australian & New Zealand journal of obstetrics & gynaecology. 1983;23(4):208–15. 6585195 10.1111/j.1479-828x.1983.tb00580.x

[28] DiGirolamo AM, Grummer-Strawn LM, Fein SB. Do perceived attitudes of physicians and hospital staff affect breastfeeding decisions? Birth. 2003;30(2):94–100. 12752166 10.1046/j.1523-536x.2003.00227.x

[29] Barnett E, Sienkiewicz M, Roholt S. Beliefs about breastfeeding: a statewide survey of health professionals. Birth. 1995;22(1):15–20. 7741946 10.1111/j.1523-536x.1995.tb00548.x

[30] Chen CH, Shu HQ, Chi CS. Breastfeeding knowledge and attitudes of health professionals and students. Acta paediatrica Taiwanica. 2001;42(4):207–11. 11550408

[31] Newcomb C.Breastfeeding and mothering in Denemark. New Beginnings. 2009;299(5–6):56–9.

[32] Scott J MR, Tappin D, Guthrie E. Breastfeeding opinions, knowledge, management practices and training of Scottish midwives. Edinburgh: Report for the Scottish Executive Health Department Chief Scientist Office; 2003.

[33] Sulaiman Z, Liamputtong P, Amir LH. The enablers and barriers to continue breast milk feeding in women returning to work. Journal of advanced nursing. 2016;72(4):825–35. doi: 10.1111/jan.12884 26749396 10.1111/jan.12884

[34] World Health Organization, Exclusive breastfeeding for six months best for babies everywhere. 15 Jan 2011. Available from: www.who.int/mediacentre/news/statements/2011/breastfeeding_20110115/en/index.html Cited Jul 2017

[35] Australian Institute of Health and Welfare. 2010 Australian National Infant Feeding Survey: Indicator Results. 20 Dec 2011. Available from: http://www.aihw.gov.au/publication-detail/?id=1073742092738.

[36] Brodribb WE, Miller YD. The impact of community health professional contact postpartum on breastfeeding at 3 months: a cross-sectional retrospective study. Maternal and child health journal. 2014;18(7):1591–8. doi: 10.1007/s10995-013-1398-3 24281850 10.1007/s10995-013-1398-3

[37] Brodribb W, Jackson C, Fallon AB, Hegney D. Breastfeeding and the responsibilities of GPs: a qualitative study of general practice registrars. Australian family physician. 2007;36(4):283–5. 17392948

[38] Meyers D. Promoting and supporting breastfeeding. American family physician. 2001;64(6):931–2. 11578031

[39] Grawey AE, Marinelli KA, Holmes AV. ABM Clinical Protocol #14: Breastfeeding-friendly physician's office: optimizing care for infants and children, revised 2013. Breastfeeding medicine: the official journal of the Academy of Breastfeeding Medicine. 2013;8:237–42. 23573799 10.1089/bfm.2013.9994

[40] Freed GL, Clark SJ, Curtis P, Sorenson JR. Breast-feeding education and practice in family medicine. The Journal of family practice. 1995;40(3):263–9. 7876784

[41] Kim HS. Attitudes and knowledge regarding breast-feeding: a survey of obstetric residents in metropolitan areas of South Korea. Southern medical journal. 1996;89(7):684–8. 8685754 10.1097/00007611-199607000-00007

[42] Goldstein AO, Freed GL. Breast-feeding counseling practices of family practice residents. Family medicine. 1993;25(8):524–9. 8405801

[43] Brodribb WE, Jackson C, Fallon AB, Hegney D. Gender and personal breastfeeding experience of rural GP registrars in Australia-a qualitative study of their effect on breastfeeding attitudes and knowledge. Rural and remote health. 2007;7(3):737 17658955

[44] Burt S, Whitmore M, Vearncombe D, Dykes F. The development and delivery of a practice-based breastfeeding education package for general practitioners in the UK. Maternal & child nutrition. 2006;2(2):91–102. 16881919 10.1111/j.1740-8709.2006.00046.x PMC6860755

[45] Holmes AV, McLeod AY, Thesing C, Kramer S, Howard CR. Physician breastfeeding education leads to practice changes and improved clinical outcomes. Breastfeeding medicine: the official journal of the Academy of Breastfeeding Medicine. 2012;7(6):403–8. 23046226 10.1089/bfm.2012.0028

