# Supplementary material for: Australian general practitioners' knowledge, attitudes and practices towards breastfeeding
Source: PLoS One. 2018 Feb 28;13(2):e0191854. doi: 10.1371/journal.pone.0191854 (PMC5830034; doi:10.1371/journal.pone.0191854)
Supplement: S1 Interview Guide — (DOCX) [file pone.0191854.s001.docx]

Thank you for you agreeing to talk to me today. My name is Orit, and I am looking at general

practitioners views, practices and knowledge of breastfeeding. Very little information has been gathered on these issues in Australia to date.

Information gathered from these interviews will hopefully be used in the future to construct a breastfeeding education program for GPs.

**Interview guide- breastfeeding attitudes and practices**

| **Topic** | **Initial broad questions** | **Possible probing questions (guide only)** |
| --- | --- | --- |
| Breastfeeding attitudes- general | Can you tell me about your views on breastfeeding in general? | - What benefits, if any, do you think breastfeeding provides? - What is the difference between breastfeeding and formula feeding? - What do you think about breastfeeding in public? - How do you think breastfeeding affects the mother and infant relationship? - How do you think breastfeeding affects the father and infant relationship? - How do you view the nutritional value and digestibility of breast milk? - What do you think about breastfeeding and women who smoke/drink? - What do you think about breastfeeding and return do work? - What are your views about weaning from breastfeeding? |
| GP's role | What do you see as your role, if any, as a general practitioner in encouraging or supporting breastfeeding women? | - What is your importance as a GP in supporting and encouraging breastfeeding? - What is the influence a GP has on a woman's decision to breastfeed - What is the role of the GP in the decision making process regarding breastfeeding method? - When do you think you should discuss breastfeeding with a patient (early pregnancy, postpartum)? - What is the effect of the gender of the GP on their ability to support breastfeeding women? |
| Personal experience | What is your personal experience with breastfeeding? | - Have you or your partner breastfed (how long, how many children)? - How would you describe your breastfeeding experience? - How has your breastfeeding experience affected the way you support breastfeeding women? |
| Breastfeeding practices- general | Can you tell me how you provide encouragement and support to breastfeeding women | - How, if at all, do you assist women with deciding on infant feeding method? - How do you encourage breastfeeding women? - How do you assist women with solving breastfeeding problems? - How if at all, do you discuss breastfeeding with women who chose to formula feed? - To whom, if at all, do you refer women with breastfeeding problems? |
| Breastfeeding- friendly practice | How, if at all, have you created a breastfeeding friendly office? | - What kind of written/verbal breastfeeding-friendly policy do you have in your office? - What resources do you have available for patients (brochures, DVDs, samples) - What graphics media related to breastfeeding do you have in your office? - What is your policy regarding breastfeeding in your waiting room? - What do you do if a mother breastfeeds during a consultation? |
